# Protocol for a cohort study of the impact of the COVID-19 pandemic on the rate and incidence of bystander cardiopulmonary resuscitation (CPR) after out-of-hospital cardiac arrest

**DOI:** 10.1186/s13049-021-00890-6

**Published:** 2021-06-21

**Authors:** Ingvild B. M. Tjelmeland, Jan Wnent, Siobhan Masterson, Jo Kramer-Johansen, Jan-Thorsten Gräsner

**Affiliations:** 1 Institute for Emergency Medicine, University Hospital Schleswig-Holstein, Kiel, Germany; 2 Division of Prehospital Services, Oslo University Hospital, Oslo, Norway; 3 Faculty of Medicine, Institute of Clinical Medicine, University of Oslo, Oslo, Norway; 4 Department of Anaesthesiology and Intensive Care Medicine, University Hospital Schleswig-Holstein, Kiel, Germany; 5 School of Medicine, University of Namibia, Windhoek, Namibia; 6 Clinical Directorate, National Ambulance Service, Health Service Executive, Galway, Ireland; 7 Discipline of General Practice, National University of Ireland Galway, Galway, Ireland

**Keywords:** COVID-19, Corona, Cardiac arrest, Out-of-hospital cardiac arrest, Registries, Bystander cardiopulmonary resuscitation, CPR

## Abstract

**Background:**

Survival after out-of-hospital cardiac arrest (OHCA) is dependent on early recognition, early cardiopulmonary resuscitation (CPR) and early defibrillation. The purpose of CPR is to maintain some blood flow until the arrival of the emergency medical services (EMS). Our concern is that the COVID-19 pandemic has had a negative effect on the number of patients who get CPR before EMS arrival. The aim of this study is to compare the incidence of bystander CPR during the pandemic with data from before the pandemic.

**Methods:**

The protocol is for a retrospective cohort study where data from existing registries will be used. All participating registries will share aggregated data from 2017 to 2020, and the study team will compare the results from 2020 to results from 2017 to 2019. Due to the General Data Protection Regulation, each participating registry will check for completeness and plausibility, and perform all aggregation of data locally. In the following analysis different registries will be considered as random samples and analysed by means of a generalized linear mixed effects model with Poisson distribution for the outcome, the population covered as offsets, and different registries as random factors.

**Discussion:**

This study does not present the prospect of direct benefit to the patient, but does provide an opportunity to gain a better understanding of the epidemiology of bystander CPR for OHCA patients during a pandemic. By comparing data during the pandemic with already collected information in established registries we believe we can gain valuable information about changes in public response to out-of-hospital cardiac arrest.

**Supplementary Information:**

The online version contains supplementary material available at 10.1186/s13049-021-00890-6.

## Background

Out-of-hospital cardiac arrest (OHCA) has a high mortality rate and is still one of the leading causes of death in the developed world [1, 2]. Survival after OHCA is dependent on early recognition, early cardiopulmonary resuscitation (CPR) and early defibrillation. The chain-of-survival concept highlights both the time-sensitive nature of interventions and also that high quality in all stages of treatment is necessary to increase survival rates [3].

The purpose of CPR is to maintain a minimum of circulation to vital organs. No CPR or CPR of poor quality does not provide the cells in the body with adequate oxygen and results in rapid cell death. For patients suffering OHCA that is not witnessed by Emergency Medical Services (EMS), the person who witnesses collapse or discovers the patient i.e., bystander, needs to start CPR. Bystander CPR has been advocated and taught since the 1960s, but implementation has proven difficult. In publications reporting data from registries worldwide, bystander CPR rates ranged from 5.5 to 70.5% [2]. In a European study bystander CPR ranged from 13 to 82% [1] and in a study from Asia the reported rate varied from 10.5 to 40.9% [4]. In Australia and New Zealand, the reported bystander rate was 41% and varied between 36 and 50% [5]. In the annual report from the United States registry of cardiac arrest (CARES registry) from 2019, bystander CPR rate was 41.2% [6]. Bystander CPR rate is not a fixed feature of a region. It can be improved by interventions such as public information and training, as shown in a recent study from Asia [7].

Dispatch-assisted CPR has been implemented in many countries the last 20 years, and has in previous studies shown to improve the bystander CPR rates [7]. However major events in society may impact on bystander CPR. When the COVID-19 pandemic hit, the American Heart Association and the European Resuscitation Council both published guidelines for resuscitation of known/suspected positive cases [8, 9]. The recommendations in these guidelines were influenced by the potential for aerosol generation as a result of performing chest compressions and airway management. The recommendations were as follows:

- Compression-only resuscitation and public-access defibrillation.
- Lay rescuers who are willing, trained and able to do so, may wish to deliver rescue breaths to children in addition to chest compressions.
- Healthcare professionals should use personal protective equipment for aerosol-generating procedures during resuscitation.
- Healthcare providers should consider defibrillation before donning aerosol generating personal protective equipment in situations where the provider assesses the benefits may exceed the risks.
- When assessing breathing in situations with suspected or confirmed COVID-19, look for normal breathing. In order to minimise the risk of infection, do not open the airway and do not place your face next to the victims’ mouth / nose.
- Lay rescuers should consider placing a cloth/towel over the person’s mouth and nose before performing chest compressions and public-access defibrillation in situations with suspected or confirmed COVID-19. This may reduce the risk of airborne spread of the virus during chest compressions.
- For children in cardiac arrest rescuers who are willing and able should also *open the airway and provide rescue breaths,* as per 2015 guidelines, knowing that this is likely to increase the risk of infection.

Dispatch centres were also recommended to screen all calls for COVID-19 symptoms in the patient and among household members in order to assess the risk of infection for first responders and EMS personnel. This information could be used to alert the responding personnel to take precautions such as donning airborne-precaution personal protective equipment (PPE). These new guidelines were often adopted for all emergency calls as knowledge of the transmission risk from pre-symptomatic and asymptomatic patients became more widespread and the need to avoid inadvertent transmission to health care providers became more pronounced. The new guidelines were not uniformly adopted between and within countries, and has been a point of controversy in some systems.

## Methods/design

### Aim

The objective of this study is to investigate the impact of the COVID-19 pandemic on the rate of bystander CPR, taking into consideration implementation of new guidelines for bystander CPR and changed dispatch protocol.

**Primary Objective**

1. What is the bystander CPR incidence per 100,000 inhabitants during the COVID-19 pandemic compared with the previous 3 years?

**Secondary Objectives**

2. What is the reported incidence of cardiac arrest during the COVID-19 pandemic compared with the previous 3 years?

### Design

This study is a retrospective cohort study, using data from existing out-of-hospital cardiac arrest registries.

During the COVID-19 pandemic different parts of the world were affected at different times and implementation of restrictions, precaution measures and changes to resuscitation guidelines occurred during different time intervals. Several studies have reported that the pandemic has had an effect on the incidence of cardiac arrest, the rate of bystander CPR and OHCA survival [10–12]. Variability is however to be expected due to the stochastic nature of rare events, and to different baselines in different registries. In order to see if the COVID-19 pandemic had had any real impact on the rate of bystander CPR, the results need to be compared with previous recordings.

In this study we will consider different registries (corresponding to different populations) as random samples from the world’s target population. We will then compare the incidence during the COVID-19 time period to the 3 preceding years (2017–19) by means of a generalized linear mixed effects model.

### Setting

#### Inclusion criteria and exclusion criteria

All patients suffering OHCA from 1st of January 2017 to 31st of December 2020 will be included. Data will be extracted from existing registries. Patients will be included irrespective of their age, gender or personal factors. The start of the COVID-19 time period will be defined as the day that a state of emergency was declared for the region/country.

New-borns in need of resuscitation at birth will not be included. In-hospital cardiac arrest patients will also not be included in this study.

#### Participating registries

All established registries that are able to process core data as described in the Utstein template of 2014 [13] are invited to participate in this study. Requirements are a written letter of intent from the registry leader/board to participate in this study, a written consent to follow this study protocol and a valid ethical approval if needed (Supplementary document 1). The participants also need to be able to do the statistical calculations according to the specified analysis plan.

Within each registry the inclusion might be the entire registry or regions only, depending on data completeness. It might also be useful to divide into smaller regions within a registry during the analysis, as the impact of COVID-19 might be very different between areas.

#### Study sites

All registries capable of delivering data as described are invited to participate.

#### Statistical analysis

In this study we will consider different registries (corresponding to different populations) as random samples from the world’s target population. We will then compare the incidence during the COVID-19 time period to the 3 preceding years (2017–19), by means of a generalized linear mixed effects model with Poisson distribution for the outcome, the population covered as offsets, and different registries as random factors.

## Discussion

This study does not present the prospect of direct benefit to the patient, but does provide an opportunity to gain a better understanding of the epidemiology of bystander CPR rates for cardiac arrest patients during a pandemic. It also gives us the opportunity to study population response to a pandemic.

### Data management

Due to the General Data Protection Regulation, each participating registry will check for completeness and plausibility, and perform all calculations locally. A statistical analysis plan will be provided by the study management team to each participating registry (Supplementary document 2). In case of inconsistencies or relevant missing data, the problem will be solved locally or in cooperation with the study management team. Inclusion and exclusion for each analysis will be defined by the study management team, including how to perform the calculations. The calculated results will then be submitted to the study management team, who will compare results from the different regions.

### Project management

- Stage 1: Establishing representatives in the study management team and the scientific committee. January 2021.
- Stage 2: Establishing contact with registries that collect core Utstein variables, March 2021.
- Stage 3: Data collection and local quality control of data, December 2021.
- Stage 4: Quality control of preliminary results, April to July 2022.
- Stage 5: Presentation of results and comparison with published results, August to October 2022.
- Stage 6: Article writing and publication, August 2022 to January 2023.

**Steering Committee (SC):**

Brian McNally

Marcus Ong

Karen Smith.

Jo Kramer-Johansen

Eirik Skogvoll

Rolf Lefering

Jan Thorsten Gräsner

**Study Management Team (SMT):**

Ingvild B. M. Tjelmeland

Jan Wnent

Siobhan Masterson

Jo Kramer-Johansen

Rolf Lefering

Jan Thorsten Gräsner

**Publication and Presentation Plans**

Authors as listed in the introduction.

Acknowledgement; all participants involved in data collection.

The results will be published in a peer reviewed journal.

## Supplementary Information

**Additional file 1.** Memorandum of understanding.

**Additional file 2.** Preparation of data and statistical analysis of data for the COVID-19 bystander rate project.

## Data Availability

Data sharing is not applicable to this protocol as no datasets were generated or analysed. Data that will be used for the study will be available from the participating registries but restrictions apply to the availability of these data, which will be used under license for the current study, and so are not publicly available.

## Abbreviations

CPR: Cardiopulmonary Resuscitation
EMS: Emergency Medical Services
OHCA: Out-of-hospital cardiac arrest
PPE: Personal Protective Equipment
SC: Steering Committee
SMT: Study Management Team

## References

[1] Grasner JT, Wnent J, Herlitz J, Perkins GD, Lefering R, Tjelmeland I (2020). Survival after out-of-hospital cardiac arrest in Europe - results of the EuReCa TWO study. Resuscitation..

[2] Kiguchi T, Okubo M, Nishiyama C, Maconochie I, Ong MEH, Kern KB, Wyckoff MH, McNally B, Christensen EF, Tjelmeland I, Herlitz J, Perkins GD, Booth S, Finn J, Shahidah N, Shin SD, Bobrow BJ, Morrison LJ, Salo A, Baldi E, Burkart R, Lin CH, Jouven X, Soar J, Nolan JP, Iwami T (2020). Out-of-hospital cardiac arrest across the world: first report from the international liaison committee on resuscitation (ILCOR). Resuscitation..

[3] Cummins RO, Chamberlain DA, Abramson NS, Allen M, Baskett PJ, Becker L, Bossaert L, Delooz HH, Dick WF, Eisenberg MS (1991). Recommended guidelines for uniform reporting of data from out-of-hospital cardiac arrest: the Utstein style. A statement for health professionals from a task force of the American Heart Association, the European resuscitation council, the Heart and Stroke Foundation of Canada, and the Australian resuscitation council. Circulation..

[4] Ong ME, Shin SD, De Souza NN, Tanaka H, Nishiuchi T, Song KJ (2015). Outcomes for out-of-hospital cardiac arrests across 7 countries in Asia: the Pan Asian resuscitation outcomes study (PAROS). Resuscitation..

[5] Beck B, Bray J, Cameron P, Smith K, Walker T, Grantham H, Hein C, Thorrowgood M, Smith A, Inoue M, Smith T, Dicker B, Swain A, Bosley E, Pemberton K, McKay M, Johnston-Leek M, Perkins GD, Nichol G, Finn J, Aus-ROC Steering Committee (2018). Regional variation in the characteristics, incidence and outcomes of out-of-hospital cardiac arrest in Australia and New Zealand: results from the Aus-ROC Epistry. Resuscitation..

[6] McNally B. 2019 Annual report CARES Cardiac Arrest Registry to Enhance Survival. 2020. https://mycares.net/sitepages/uploads/2020/2019_flipbook/index.html?page=20;. Access 26 Feb 2021.

[7] Blewer AL, Ho AFW, Shahidah N, White AE, Pek PP, Ng YY, Mao DR, Tiah L, Chia MYC, Leong BSH, Cheah SO, Tham LP, Kua JPH, Arulanandam S, Østbye T, Bosworth HB, Ong MEH (2020). Impact of bystander-focused public health interventions on cardiopulmonary resuscitation and survival: a cohort study. Lancet Public Health.

[8] Nolan JP, Monsieurs KG, Bossaert L, Bottiger BW, Greif R, Lott C (2020). European resuscitation council COVID-19 guidelines executive summary. Resuscitation..

[9] Perkins GD, Morley PT, Nolan JP, Soar J, Berg K, Olasveengen T, Wyckoff M, Greif R, Singletary N, Castren M, de Caen A, Wang T, Escalante R, Merchant RM, Hazinski M, Kloeck D, Heriot G, Couper K, Neumar R (2020). International liaison committee on resuscitation: COVID-19 consensus on science, treatment recommendations and task force insights. Resuscitation..

[10] Marijon E, Karam N, Jost D, Perrot D, Frattini B, Derkenne C, Sharifzadehgan A, Waldmann V, Beganton F, Narayanan K, Lafont A, Bougouin W, Jouven X (2020). Out-of-hospital cardiac arrest during the COVID-19 pandemic in Paris, France: a population-based, observational study. Lancet Public Health.

[11] Lai PH, Lancet EA, Weiden MD, Webber MP, Zeig-Owens R, Hall CB, et al. Characteristics Associated With Out-of-Hospital Cardiac Arrests and Resuscitations During the Novel Coronavirus Disease 2019 Pandemic in New York City. JAMA Cardiol. 2020;5:1154-63. 10.1001/jamacardio.2020.2488 PMC7305567 32558876

[12] Baldi E, Sechi GM, Mare C, Canevari F, Brancaglione A, Primi R, Klersy C, Palo A, Contri E, Ronchi V, Beretta G, Reali F, Parogni P, Facchin F, Bua D, Rizzi U, Bussi D, Ruggeri S, Oltrona Visconti L, Savastano S, Lombardia CARe Researchers (2020). Out-of-hospital cardiac arrest during the Covid-19 outbreak in Italy. N Engl J Med.

[13] Perkins GD, Jacobs IG, Nadkarni VM, Berg RA, Bhanji F, Biarent D, et al. Cardiac Arrest and Cardiopulmonary Resuscitation Outcome Reports: Update of the Utstein Resuscitation Registry Templates for Out-of-Hospital Cardiac Arrest: A Statement for Healthcare Professionals From a Task Force of the International Liaison Committee on Resuscitation (American Heart Association, European Resuscitation Council, Australian and New Zealand Council on Resuscitation, Heart and Stroke Foundation of Canada, InterAmerican Heart Foundation, Resuscitation Council of Southern Africa, Resuscitation Council of Asia); and the American Heart Association Emergency Cardiovascular Care Committee and the Council on Cardiopulmonary, Critical Care, Perioperative and Resuscitation. Resuscitation. 2015;96:328–40. 10.1016/j.resuscitation.2014.11.002 25438254

