# Supplementary material for: Protocol for a cohort study of the impact of the COVID-19 pandemic on the rate and incidence of bystander cardiopulmonary resuscitation (CPR) after out-of-hospital cardiac arrest
Source: Scand J Trauma Resusc Emerg Med. 2021 Jun 21;29:82. doi: 10.1186/s13049-021-00890-6 (PMC8215481; doi:10.1186/s13049-021-00890-6)
Supplement: Supplementary file 1 — Additional file 1. Memorandum of understanding. [file 13049_2021_890_MOESM1_ESM.docx]

Memorandum of understanding

A cohort study of the impact of the COVID-19 pandemic on the rate and incidence of bystander cardiopulmonary resuscitation (CPR) after out-of-hospital cardiac arrest

**Memorandum of Understanding between the Steering Committee**

(Represented by the Chief Investigator Ingvild B. M. Tjelmeland)

and

**NAME**

(Acting as coordinator for the study related the impact of the COVID-19 pandemic on the rate and incidence of bystander cardiopulmonary resuscitation (CPR))

Dear colleague,

This document outlines your responsibilities as Coordinator for the bystander incidence during COVID-19 study. By signing this ***Memorandum of Understanding*** (MoU) you agree to act as the Coordinator. This role covers several tasks:

There will only be one coordinator in every registry. Coordinators are responsible for ensuring that mandatory approvals (e.g. ethics approval) exist; communication with the participating registry or registries; measures to generate good data quality, supervision of data collection and complete transmission of the data from their registry.

The coordinator is an outstanding expert in the field of resuscitation and represents the registry in relation with others (participating registries, EMS, press, public, others). S/he should therefore be committed to the idea, aim and detailed contents of the Study.

(1) You must:

- Firstly; investigate whether a local, regional or national registry meets the basic criteria for participation in this study. Only data from registries that can meet the basic criteria can be included in the Study.
- Obtain ethical approval in your own country or organise a documented waiver and forward this document to the Study Management Team (SMT) when requested
- Obtain a written letter of intent to participate in the Study from any local, regional or national registry and forward this document to the SMT when requested. This letter should also state that the registry intends to follow the Study protocol
- Ensure that there is written approval from all participating EMS agencies permitting the submission of data generated by EMS to the Study

(2) Communication issues:

You will be the primary contact person for all questions concerning the Study in your registry. Should difficult questions and problems arise, these may be forwarded to the SMT, who may refer to the Steering Group if necessary.

(3)

With regard to data protection, you must ensure that data – whether computer or paper-based – is handled according to your national laws.

(4)

With regard to data quality you are responsible for:

- Ensuring that data is anonymised before being submitted to the Study
- Quality control, including ensuring reliability of the completeness of data and data validation
- Avoiding multiple submission of patients’ data (especially if more than one registry serves a specific population)
- Once data is submitted, if the SMT have any questions in relation to any of the data, you are responsible for liaising with the data provider(s) to answer these questions
- Some registries perform missing case and missing data searches to ensure the completeness of their data. It is acknowledged that some cases or data for the data collection period may be identified by individual registries after the submission deadline. The SMT may request the Coordinator to submit such data to the Study.

(5)

You must ensure submission of the data for your registry on time. It is your responsibility to submit all national data (in electronic format) to the SMT within the defined period.

(6)

It is your duty to ensure maintenance of confidentiality data and preliminary study results in your country throughout the study period.

(7)

The Study combines several registries. To ensure the success of this international project it is essential that participants adhere to the study protocol and associated documents.

The Steering Committee has the right to refuse data from any Coordinator especially if data is not delivered in the complete, reliable, high quality manner within the requested period of time.

*Place, date, signatures*
