# Supplementary material for: Protocol for a cohort study of the impact of the COVID-19 pandemic on the rate and incidence of bystander cardiopulmonary resuscitation (CPR) after out-of-hospital cardiac arrest
Source: Scand J Trauma Resusc Emerg Med. 2021 Jun 21;29:82. doi: 10.1186/s13049-021-00890-6 (PMC8215481; doi:10.1186/s13049-021-00890-6)
Supplement: Supplementary file 2 — Additional file 2. Preparation of data and statistical analysis of data for the COVID-19 bystander rate project. [file 13049_2021_890_MOESM2_ESM.docx]

Preparation of data and statistical analysis of data for the COVID-19 bystander rate project

# Inclusion criteria for patients:

All patients suffering OHCA, from 1^st^ of January 2017 to 31^st^ of December 2020. Patients are to be included irrespective of their age, gender or personal factors. All causes of cardiac arrest should be included.

# Exclusion criteria:

New-borns in need of resuscitation at birth will not be included. In-hospital cardiac arrest patients will also not be included in this study.

# Dates needed for division of data set

Date when there was declared a state of emergency

Date of first COVID-19 related death (if any)

# Statistical analysis

1. Data from 2017 to 2020 should be extracted from the registry data. The data set needs to be divided into yearly cohorts and for 2020 divided into two data sets. Data set 1 for 2020: All patients from 2017 to the date of emergency in 2020. Data set 2 for 2020: All patients during the pandemic period (first date in a state of emergency until 31^st^ of December 2020.
2. Calculate incidence during the COVID-19 era by multiplying the total number of patients resuscitated by bystander and/or EMS by 365 and dividing by the number of days after the declaration of a state of emergency. This number is the presumed number of cardiac arrests that would have occurred if the state of emergency was declared for all of 2020.
3. The number of inhabitants for the relevant period in 2020 needs to be calculated by multiplying the inhabitants for the entire year by the number of days of inclusion and dividing by 365.
4. The control period of 2017 to 2019, including the patients from 2020 before the state of emergency, needs to be calculated using the same formula as for incidence during the COVID-19 pandemic. The same applies for the population during this period.
5. The incidence of cardiac arrest patients is calculated by dividing the presumed number of patients with the calculated population and multiplying with 100,000.
6. Calculate the incidence of bystander CPR, excluding all EMS witnessed cases, using the same formula.
7. Calculate the rate of bystander CPR by dividing the number of patients receiving CPR before EMS arrival with the total number of included patients, minus EMS witnessed cardiac arrests

# Number of patients to be included

| **Data variable** | **2017** | **2018** | **2019** | **Before 2020** | **After 2020** |
| --- | --- | --- | --- | --- | --- |
| Population in catchment area | Total population | Total population | Total population | Total population * (number of days before the pandemic) / 365 (Population1_2020) | Total population * (number of days during the pandemic) / 365 (Population2_2020) |
| Patients resuscitated by bystander, first responder or EMS | Number of patients(1A) | Number of patients (1B) | Number of patients(1C) | Number of patients (1D) | Number of patients (1E) |
| Number of cardiac arrests witnessed by EMS | Number of patients (2A) | Number of patients (2B) | Number of patients (2C) | Number of patients (2D) | Number of patients (2E) |
| Number of patients resuscitated by bystander before EMS arrival | Number of patients (3A) | Number of patients (3B) | Number of patients (3C) | Number of patients (3D) | Number of patients (3E) |
| Number of patients resuscitated by EMS | Number of patients (4A) | Number of patients (4B) | Number of patients (4C) | Number of patients (4D) | Number of patients (4E) |
| Mean age | Years (SD) | Years (SD) | Years (SD) | Years (SD) | Years (SD) |
| Number of patients under 14 | Number of patients (5A) | Number of patients (5B) | Number of patients (5C) | Number of patients (5D) | Number of patients (5E) |
| Number of patients that are male | Number of patients (6A) | Number of patients (6B) | Number of patients (6C) | Number of patients (6D) | Number of patients (6E) |

*Table 1: Number of cardiac arrest patients included in each group from 2017 to 2020. For 2020 the number of days is divided into two groups, before and after a state of emergency was declared.*

# Calculations

| **Data variable** | **2017** | **2018** | **2019** | **Before 2020** | **After 2020** |
| --- | --- | --- | --- | --- | --- |
| Incidence of cardiac arrest | 1A/ (total population) * 100,000 | 1B/ (total population) * 100,000 | 1C/ (total population) * 100,000 | 1D/(Population1_2020) * 100,000 | 1E/(Population2_2020) * 100,000 |
| Incidence of EMS witnessed cardiac arrests | 2A/ (total population) * 100,000 | 2B/ (total population) * 100,000 | 2C/ (total population) * 100,000 | 2D/(Population1_2020) * 100,000 | 2E/(Population2_2020) * 100,000 |
| Incidence of bystander CPR | 3A/ (total population) * 100,000 | 3B/ (total population) * 100,000 | 3C/ (total population) * 100,000 | 3D/ (Population1_2020* 100,000 | 3E/(Population2_2020) * 100,000 |
| Rate of bystander CPR | 3A/(1A-2A) * 100 | 3B/(1B-2B) *100 | 3C/(1C-2C) * 100 | 3D/ (1D-2D) * 100 | 3E/ (1E-2E) *100 |
| Incidence of EMS treated patients | 4A/ (total population) * 100,000 | 4B/ (total population) * 100,000 | 4C/ (total population) * 100,000 | 4D/(Population1_2020) * 100,000 | 4E/(Population2_2020) * 100,000 |

*Table 2: Calculation of incidence rates based on the number of reported patients in each group from 2017 to 2020. For 2020 the number of days is divided into two groups, before and after a state of emergency was declared.*

# Sharing of data with the project management team

Data needs to be shared with the study management team on time and needs to be of good quality in accordance with the memorandum of Understanding.
